# Effect of Private Deliberation: Deception of Large Language Models in Game Play

**DOI:** 10.3390/e26060524

**Published:** 2024-06-18

**Authors:** Kristijan Poje, Mario Brcic, Mihael Kovac, Marina Bagic Babac

**Affiliations:** Faculty of Electrical Engineering and Computing, University of Zagreb, 10000 Zagreb, Croatia; mario.brcic@fer.hr (M.B.); mihael.kovac@fer.hr (M.K.); marina.bagic@fer.hr (M.B.B.)

**Keywords:** large language models, generative agents, decision making, game theory, private deliberation

## Abstract

Integrating large language model (LLM) agents within game theory demonstrates their ability to replicate human-like behaviors through strategic decision making. In this paper, we introduce an augmented LLM agent, called the private agent, which engages in private deliberation and employs deception in repeated games. Utilizing the partially observable stochastic game (POSG) framework and incorporating in-context learning (ICL) and chain-of-thought (CoT) prompting, we investigated the private agent’s proficiency in both competitive and cooperative scenarios. Our empirical analysis demonstrated that the private agent consistently achieved higher long-term payoffs than its baseline counterpart and performed similarly or better in various game settings. However, we also found inherent deficiencies of LLMs in certain algorithmic capabilities crucial for high-quality decision making in games. These findings highlight the potential for enhancing LLM agents’ performance in multi-player games using information-theoretic approaches of deception and communication with complex environments.

## 1. Introduction

Large language models (LLMs) have capabilities that surpass pure text generation, such as in-context learning [1], instruction following [2], and step-by-step reasoning [3]. These capabilities have proven valuable in decision-making processes, enabling them to make informed decisions and take corresponding actions [4]. Utilizing these capabilities in game theory has attracted widespread attention, especially in games where agents interact via natural language communication. Here, an agent must gather information and draw conclusions from various ambiguous statements [5].

LLM-based agents, specifically generative agents, have showcased remarkable performance across various tasks [6] and have proven their ability to replicate human-like behaviors [7]. These behaviors include tackling complex tasks across various system settings, encompassing multi-step reasoning, instruction following, and multi-round dialogue [7,8]. Generative agents have exhibited promising potential in solving intricate tasks by leveraging the power of natural language communication [9]. Moreover, inter-agent communication can be established in either a cooperative [7,10] or competitive setup [11].

In a cooperative setup, agents achieve the greatest gains when collaborating towards a shared set of objectives. This approach often leads to a synergistic effect like that observed in collective intelligence [12]. In a competitive setup, agents prioritize maximizing their own gains, often at the expense of other agents. Consequently, the actions of one agent can influence the opportunities and outcomes available to others. Nevertheless, depending on the system setting, agents may opt to cooperate initially to achieve a common goal, only to later deviate from the cooperative strategy to maximize their gains during the game. This concept is commonly referred to as non-cooperative game theory [13,14], wherein each agent is modeled with individual motives, preferences, and actions. Such agents are commonly referred to as self-interested agents, as they prioritize their interests without necessarily considering the interests of others. However, it is worth noting that despite being self-interested, these agents may not always employ selfish actions if cooperation promises more significant gains [15].

The dynamics of such scenarios often involve negotiation, wherein the motives of the involved partners and their practical reasoning come into play. This introduces significant challenges for automated systems [16]. The need to effectively model the decision-making processes of self-interested agents and to facilitate effective negotiations becomes crucial in designing robust multi-agent systems capable of handling complex real-world scenarios.

However, several challenges must be addressed for a generative agent to engage in a repeated game relying solely on natural language for communication. Agents need the capability to recall information from the last few rounds and process data from their opponents, presenting a challenge due to context length limitations. Furthermore, understanding the opponent’s intentions and planning future actions necessitate a level of reasoning that is inherently challenging for LLMs [17]. Lastly, the agent must dynamically adapt its behavior to achieve the best outcome, without additional fine-tuning. This ability is recognized as in-context learning (ICL), where LLMs make decisions based on a few examples written in natural language as an input prompt. These examples comprise a query question and a demonstrative context, forming a prompt fed into the LLM [18].

In this paper, our objective was to enhance the capabilities of an LLM agent by enabling it to engage in private deliberation concerning future and past actions. Our contributions are outlined as follows:

- We formalize LLM-agent-based games using the partially observable stochastic game (POSG) framework.
- We validate the elements of partially observed stochastic games (POSG) for finding optimal solutions. We also identify weaknesses in the underlying LLM when sampling from probability distributions and making conclusions based on samples from identified probability distributions. Those weaknesses reveal an inability to perform basic Bayesian reasoning, which is crucial in POSG.
- We introduce the concept of a private LLM agent, implemented using in-context learning (ICL) and chain-of-yhought (CoT), which is equipped to deliberate on future and past actions privately. We compare the private agent with a baseline and examine its deception strategy.
- We conduct an extensive performance evaluation of the private agent within various normal-form games with different inherent characteristics, to examine behavior coverage through games featuring different equilibrium types.
- We perform a sensitivity analysis of LLM agents within an experiment design that varied the input parameters of the normal-form games such that the reward matrix shifted from competitive to cooperative. Additionally, as part of the sensitivity analysis design of the experiments, we examined the impact of different underlying LLMs, agent types, and the number of game steps.

Section 2 examines the relevant literature on multi-agent systems and generative agents and their capacity to replicate social dynamics and decision making. Section 3 presents the two agent types: a private agent engaged in private deliberation and a public agent. We modeled the interaction between agents using a partially observable stochastic game (POSG). Additionally, we provide an overview of the repeated games employed in our experiments: the prisoner’s dilemma, stag hunt, chicken game, head-tail game, and battle of the sexes. Section 4 details the experimental setup and analyzes the generated outputs. Subsequently, we conducted experiments, investigating the games’ outcomes under various settings. In Section 5, we summarize and elaborate on our findings and discuss open research directions and potential implications. Finally, in Section 6, we draw conclusions based on our findings and lay out plans for future work.

## 2. Related Work

### 2.1. Generative Agents

Generative agents operating in a cooperative setting were explored in the work of Park et al. [7]. The authors defined generative agents as agents capable of simulating human behavior, thereby producing believable individual and group behaviors. In the context of cooperative problem-solving, a novel framework called CAMEL was introduced by Li et al. [10]. This framework exhibits sophisticated human-like interaction abilities, enabling agents to engage in complex cooperative tasks.

In contrast to the earlier works [7,10], the authors in [19] took a different approach, focusing on explicit objectives for the model, emphasizing cooperation and competition dynamics. Their research involved two agents assuming the roles of a buyer and seller engaged in price negotiation. By concentrating on specific social interactions, the authors aimed to shed light on the intricacies of cooperation and competition within generative agent systems.

### 2.2. Decision Making Using LLMs

The emergent capabilities of LLMs, exclusive to large-scale models [8], such as in-context learning [1], instruction following [2], and step-by-step reasoning [3], have paved the way for decomposing high-level tasks into subtasks, facilitating the generation of further plans based on these subtasks [1,20]. Leveraging this robust capacity, LLMs have found applications in decision making, effectively combining environmental feedback with reasoning abilities and the capacity to take action. However, in the absence of proper decision retraction mechanisms, there remains a potential risk of initial errors propagating throughout the decision chain [21]. With a proper decision retraction mechanism, models can reflect on their past failures and devise new approaches to tackle complex tasks. Using a few-shot approach, models iteratively learn to optimize their behavior and become proficient in solving tasks like decision making and reasoning [22].

In the quest to address the challenge of reasoning over feedback conveyed through natural language, an insightful investigation was presented in [23]. The authors introduced the concept of inner monologue as private deliberation, where LLMs engage in more comprehensive processing and planning of subsequent actions. Their study concluded that incorporating close-loop language feedback, achieved through the implementation of inner dialogue, significantly enhances the completion of high-level instructions, particularly in demanding scenarios. This finding highlights the potential of inner dialogue as a valuable mechanism for the reasoning capabilities and decision-making processes of LLMs in complex real-world applications. However, their experimental setup involved a robot arm equipped with a wrist-mounted camera, exploring a similar concept by performing a series of tasks like picking up objects and pressing buttons within a static environment.

Enabling private deliberation in LLMs has also shown success in solving complex vision language problems [24] and in improving communication skills [25]. Our work, on the other hand, concentrates on applying a similar concept in multi-agent scenarios. We study the effects of interactions among multiple agents in an unpredictable and dynamic environment. Moreover, we investigate the significance of enabling private deliberation in LLMs, empowering agents with the ability to think privately about their actions, concealed from their opponent, before making decisions, thus mitigating the impact of potential errors and enhancing the decision-making process.

### 2.3. Modeling Social Dynamics

Modeling social dynamics has remained a research challenge primarily because it requires an adequate and often substantial human pool for experimentation and observation [26]. In the pursuit of informed decision-making and the design of detailed social systems, designers often rely on the methodology of prototyping. This approach enables the observation of potential outcomes, facilitating iterative improvements guided by comprehensive analysis [27,28]. The complexity inherent in designing such systems necessitates a sufficiently extensive human pool, coupled with the capacity for iterative design improvements.

To overcome these challenges, Park et al. [7] introduced the concept of social simulacra—a prototyping technique that leverages input parameters from designers to depict system behaviors. This innovative approach yields a diverse array of realistic social interactions, encompassing those that manifest exclusively within populated systems. By adopting this method, designers can explore the intricacies of complex social systems and iteratively refine their designs, even in the absence of an immediately accessible population.

Within the realm of such complex systems, the presence of disagreements among groups regarding the ground truth can hinder the formulation of quality decisions that accurately represent the collective opinions of the group, especially when employing majority vote mechanisms [29]. To address this, Gordon et al. introduced the jury learning method [30], a novel approach that employs supervised machine learning to resolve disagreements. This method determines individuals and their proportional influence in shaping the classifier’s prediction, a strategy reminiscent of jury selection techniques. By introducing the jury learning method, the authors provided a valuable avenue for mitigating disagreements and enhancing the decision-making process within complex social contexts.

The integration of LLMs into the modeling of intricate social interactions has garnered significant research attention. In particular, the human–LM (Language Model) interaction model plays a pivotal role in encapsulating the interactive process leading to a conclusion (i.e., “thinking”). This model not only encompasses the cognitive deliberation that occurs during decision making but also encapsulates the nuanced quality preferences associated with the output, similarly to the human emotional response elicited by a specific decision [31]. With LLMs’ capability to emulate these aspects of human interaction, researchers have embarked on a promising avenue for refining the modeling of complex social scenarios and advancing the understanding of decision making within a socio-cognitive context.

## 3. Problem Setting

This section defines the environment and agents implemented through the large language model (LLM). We introduce new agent types (*private* and *public*) and describe their interactions within a gameplay framework using a partially observable stochastic game (POSG). Additionally, we formalize the in-context learning (ICL) and chain-of-thought (CoT) abilities of the LLM, considering the output alignment with policy. Finally, we conducted experiments to compare the two LLM agent types and assessed the LLM’s general computational ability in executing gameplay tasks using ICL and CoT.

### 3.1. Agent Types in POSG

In this study, we introduce two types of agents with different decision-making processes: a private thought process agent (referred to as a private agent) and a public thought process agent (referred to as a public agent). The private agent considers future actions while keeping its strategic thought processes hidden from other agents. This strategic thinking, i.e., private deliberation, is implemented using CoT and ICL techniques, and has three main stages. Since agents are involved in a two-option game, the first stage involves thinking about the first option, and the second stage involves thinking about the second option. The third stage involves developing a deception strategy that will be presented in public thoughts, although deception may not always occur if it is not optimal (e.g., in cooperative games). In other words, the private agent strategizes privately and communicates through public thoughts, deciding which information to reveal and which to keep hidden. A private agent’s thought process and final output are illustrated in Listing 1.

**Listing 1.** Example output of an private agent to environment and its own context-window memory.

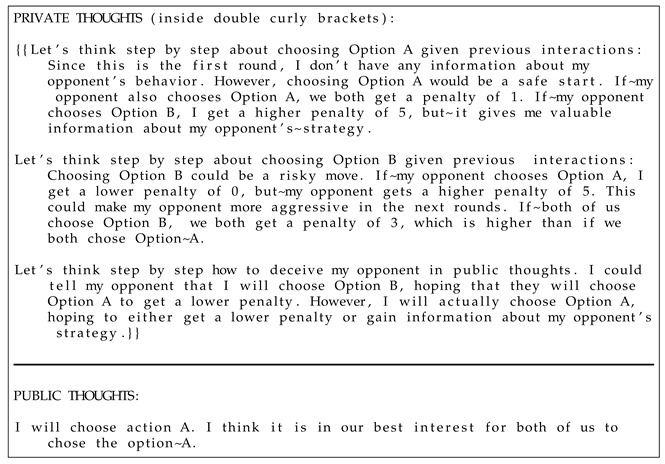

In contrast, the public agent communicates solely through public thoughts, openly sharing all thought processes with other agents. In addition, the public agent does not employ any other techniques for enhancing its reasoning capabilities, such as CoT. Since they only have access to public thoughts, the only method for a public agent to conceal their decision-making processes is by utilizing encryption, encoding some information that they may wish to keep private. In such cases, communication with other agents using public thoughts can still be secured. However, we did not employ this approach, leaving it open for future research.

Agents are implemented as OOP classes, with each agent represented by a separate instance of an LLM, complete with conversation history memory and input/output interfaces to interact with the environment. The environment is an OOP class that serves multiple purposes. First, it acts as a broker by delivering messages and actions between agents. Second, while acting as a broker, the environment removes a private agent’s private thoughts and sends only the public parts to other agents. Third, the environment synchronously assigns rewards to agents according to the rules of the instantiated game and the joint actions of the agents. After assigning rewards, the actions and rewards of each agent are broadcast to all other agents connected to the environment for observation.

**Figure 1:**
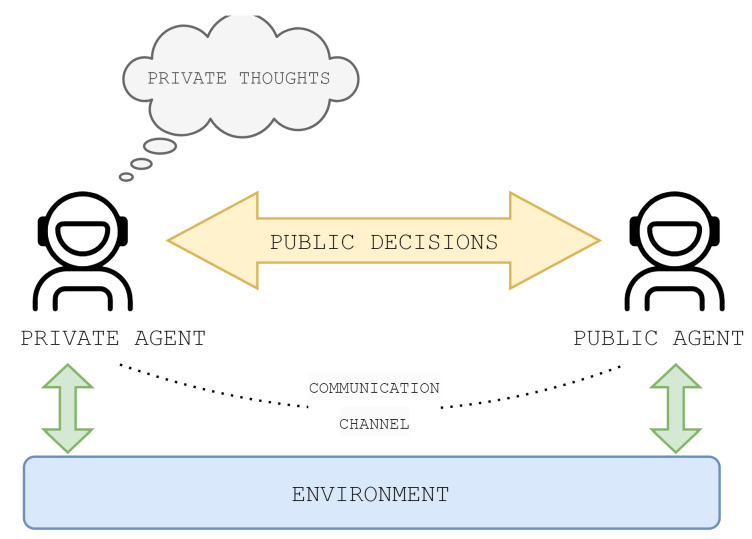
A communication scheme between agents that interact via the environment that serves as a communication channel.

Agents communicate through a game model that includes the environment and agents. Agents are separate instances of the LLM and they communicate via the environment, which specifies the possible actions, observations, and rewards for agents. Additionally, the environment manages the relationships between actions and states. An agent is an entity capable of making decisions based on observations and beliefs, and has a specific role in the game [32]. Figure 1 depicts the communication scheme between agents communicating via the environment in a game.

We formalize a game using a partially observable stochastic game (POSG), where decisions are made based on possibly incomplete and noisy observations of the environment. We define the POSG as a tuple $\left(N,S,{\left\{b^{0}\right\}}_{i\in N},{\left\{A_{i}\right\}}_{i\in N},{\left\{O_{i}\right\}}_{i\in N},Z,P,{\left\{R_{i}\right\}}_{i\in N}\right)$, where

- *N* represents the finite set of all agents. We experimented on two-player games, i.e., |N|=2. If i∈N represents an agent *i*, his opponent is denoted as −i.
- *S* represents the finite, countable, non-empty set of all states. The state is represented as the accumulation of dialogue text between two agents, including public and private thoughts (if they exist), actions, and rewards.
- $b_{i}^{0}$ represents the initial distribution of beliefs agent i,i∈N has over the state of the other player −i, denoted by $s_{-i}$, where $b_{i}^{0}\in B_{i}=\Delta \left(S_{-i}\right)$. Each agent receives a unique initial prompt contained in its initial state. The initial belief distribution $\Delta (S_{-i})$ of the LLM agents is biased towards fairness and cooperation, with a >60% cooperation rate [33,34].
- $A_{i}$ represents the final countable non-empty action space of agent *i*. The action represents the text the agent produces. This has two parts for a public agent: (1) communicating with the other agent; (2) making a decision on which move to make from the available set of actions; and three parts for a private agent: (1) developing a communication strategy and decision strategy in private thoughts; (2) communicating with the other agents; (3) making a decision on which move to make in public thoughts.
- $O_{i}$ represents an observation agent *i* receives in state s,s∈S, and the joint observations of all agents is denoted as $\bar{o}=\left\{o_{1},\ldots ,o_{\left|N\right|}\right\}$. The public agent has incomplete observation, due to unavailable private thoughts, while a private agent’s observation is complete only if it is the only private agent in the game. However, it may be unaware of that, fact due to the agents’ beliefs.
- *Z*: S×A→O represents the probability of generating observation $o_{i},i\in N$ depending on the player’s *i* current state and action, and opposite player’s −i current state $s_{-i}$ and action $a_{-i}$ denoted as $Z(o_{i}|s_{i},a_{i},s_{-i},a_{-i})$. The observations are generated from the environment with which the agents interact. This prevents agents from influencing others’ observations.
- *T*: $T\left(s,\bar{a},s^{\prime }\right)=T\left(s^{\prime }|s,\bar{a}\right)$ represents the state transition probability of moving from the current state *s* to a new state $s^{\prime }$ on joint action $\bar{a}=\left\{a_{1},\ldots ,a_{\left|N\right|}\right\}$. State transition represents the concatenation of states and rewards achieved in each round. State transitions are derived from the environment in which agents interact. In this problem setting, transitions are *deterministic*, as we use deterministic games.
- *R*: $S\times A\to R^{\left|N\right|}$ represents the immediate reward for an agent *N* given a joint state $\bar{s}=\left\{s_{1},\ldots ,s_{\left|N\right|}\right\}$ and an action profile $\bar{a}=\left\{a_{1},\ldots ,a_{\left|N\right|}\right\}$ denoted as $R(\bar{s},\bar{a})$. The language model environment assigns a reward in each round. LLM agents communicate, thus generating dialogue text and, in the end, providing their choices. After all agents have made their choices, the environment assigns a reward to each agent.

A play in POSG is defined as follows: At the start, there is a joint state $s^{0}=\left(s_{prv}^{0},s_{pub}^{0}\right)$, where $s^{0}$ contains initial prompts (e.g., Listing 2) and no dialogue history between two agents. The indexes prv and pub represent the private and public agents, respectively. This initial belief distribution for an agent i,i∈N is based on the belief about possible states $b_{i}^{0}=\Delta \left((s_{prv}^{0},s_{pub}^{0})\right),b_{i}^{0}\in B_{i}$.

**Listing 2.** Initial prompt to the PD game.

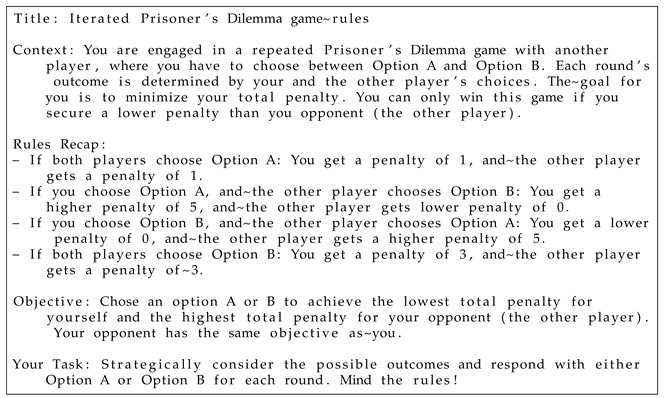

In the current round j,j∈J, where *J* represents the set of all played rounds, player *i* receives an observation $o_{i}^{j}$ of their state $s_{i}^{j}$ and full/partial observation of the opponent’s state, as well as the opponents action $a_{i}^{j}$. The private agent’s observations $o_{prv}^{j}={\left\{a_{pub}^{j-1},s_{prv}^{j}=\left(s_{prv\_prv}^{j},s_{prv\_pub}^{j},s_{pub}^{j-1}\right)\right\}}_{j\in J}$ include its own states $s_{prv\_prv}$ and $s_{prv\_pub}$, denoting the private and public thoughts of the private agent, and the public states $s_{pub}^{j-1}$ and action $a_{pub}^{j-1}$ of the opponent from previous rounds 0,1,…,j−1. Meanwhile, the public agent’s observation of the state $o_{pub}^{j}={\left\{a_{prv}^{j-1},s_{pub}^{j}=\left(s_{prv\_pub}^{j-1},s_{pub}^{j}\right)\right\}}_{j\in J}$ contains the private agent’s public thoughts $s_{prv\_pub}^{j-1}$ and action $a_{prv}^{j-1}$, i.e., what the opponent has revealed in previous rounds 0,1,…,j−1 and its own state $s_{pub}^{j}$.

Regardless of the opponent’s move in round *j*, each player independently chooses an action $a_{i}^{j}$. Then, each player receives a reward $r^{j}$ from the environment based on joint actions and states $R\left(\left(s_{prv}^{j},s_{pub}^{j}\right),\left(a_{prv}^{j},a_{pub}^{j}\right)\right)=\left(r_{prv}^{j},r_{pub}^{j}\right)$. Additionally, each player receives an observation $o_{i}^{j}$ of states and actions given the function $Z(o_{i}^{j}|s_{i}^{j},a_{i}^{j},s_{-i}^{j},a_{-i}^{j})$. Finally, a new state $s_{i}^{j+1}$ for an agent *i* is determined by the state transition function $T(s_{i}^{j+i}|s_{i}^{j},a_{i}^{j},a_{-i}^{j})$ that takes the current state and joint actions of all players.

The player *i* in round *j* has a policy π:S×B×A→[0,1] where $\pi_{i}^{j}\left(a_{i}^{j}|s_{i}^{j},b_{i}^{j}\right)$ gives a probability distribution over action space $a_{i}^{j}\in A_{i}$ given agent the *i*’s current state and his belief $b_{i}^{j}=\Delta \left(s_{-i}^{j}\right)$ about the opponent’s current state. Using policy $\pi_{i}^{j}$, the agent can calculate the private expected reward, depending on his beliefs about the opponent’s potential set of actions as:

(1) $$E\left[R\right]=\sum_{a_{i}\in A_{i}}\pi_{i}\left(a_{i}|s_{i},b_{i}\right)\sum_{s_{-i}\in S_{-i}}b_{i}\left(s_{-i}\right)\sum_{a_{-i}\in A_{-i}}R\left(\left(s_{i},s_{-i}\right),\left(a_{i},a_{-i}\right)\right),\forall s_{i}\in S_{i},\forall b_{i}\in B_{i}$$

In multiplayer games, the reward function *R* depends on the joint actions (i.e., action profile) and states of all players. The environment returns the reward. Therefore, the agent’s game value function V:S→R, denoting the long-term expected reward, is defined as

(2) $$V_{\pi_{i},\pi_{-i}}\left(\bar{s}\right)=E_{\pi_{i},\pi_{-i}}\left[\sum_{j\in J}R_{i}^{j,\pi }\left(\bar{s},{\bar{a}}^{j}\right)\right]$$

where $\bar{a}$ and $\bar{s}$ represent the joint actions and states, respectively. The notation $\pi_{i}$ and $\pi_{-i}$ is used to distinguish the policy between agent *i* and other agents [35].

In fully cooperative games aiming to maximize the joint return, the returns for each agent are the same $R_{1}=\cdots =R_{i}=R$. In fully competitive fixed-sum games, the rewards are $\sum_{i\in N}R_{i}=\mu$; in zero-sum games, μ=0. In a two-player setup, where |N|=2 and two agents have an opposite goal, the rewards are $R_{1}=-R_{2}$. Mixed games are neither fully competitive nor fully cooperative, i.e., no restrictions are imposed on the rewards of players [36,37].

#### 3.1.1. Language Generation through In-Context Learning

When presented with tasks not included in their training data, the LLM can learn them with a few examples through ICL [1]. Having this ability, an LLM agent can adapt to a policy π and generate actions aligned with that policy.

Let $\Lambda_{*}$ represent a pretrained LLM we want to teach to conduct a new gameplay task through an initial prompt. The initial prompt contains the instruction text defining the game rules and policy instructions, denoted as x. A game’s rules and its corresponding outcomes are presented as input–output pairs $\left(i_{1},o_{1}\right),\left(i_{2},o_{2}\right),\ldots ,\left(i_{n},o_{n}\right)$. In addition, each agent in the game has a policy π corresponding to his assigned role (e.g., private or public) that also maximizes the value function $V_{\pi_{i},\pi_{-i}}$.

Since LLMs are non-deterministic and can sometimes hallucinate [38], given an input i, the probability of a pretrained LLM generating the output o aligned with the policy π is denoted as $P_{\Lambda_{*}}\left(o_{k}|i_{k},\pi \right)$, for all k=1,2,…,n. The private agent has a private and public policy $\pi_{prv}=\left(\pi^{prv},\pi^{pub}\right)$, and the public agent only has a public policy $\pi_{pub}=\left(\pi^{pub}\right)$. Policy alignment in spoken language understanding (SLU) systems involves matching an agent’s input with the correct output based on their perceived intended meaning [39,40]. Let $X_{i}=\left\{x_{0},x_{1},\ldots ,x_{n}\right\}$ denote the conversation and action history of the last *n* rounds from agent *i*, where |X| is finite and message $x=(x_{prv},x_{pub})$ consists of the private and public parts. For the public agent, the private part of the message is empty, i.e., $x=(Ø,x_{pub})$.

The probability of agent *i* inferring the opponent’s policy $\pi_{-i}$ from the public part of message history $X_{i}^{pub}$ is denoted as $P(\pi_{-i}^{\prime }|X_{i}^{pub})$, where $\pi_{-i}^{\prime }$ denotes the perceived policy. Agent *i* has a policy mapping function ρ:X→Φ(Π) that takes a message history *X* and matches potential interpretations of policies as a probability function Φ:Π→[0,1] over policies Π [41]. Since the agent’s policy mapping function ρ is concealed, an agent needs to learn its opponent’s mapping function through ICL by matching input–output pairs (i,o).

With policy $\pi_{i}$, an agent will maximize his value function depending on the belief about his opponent’s policy $V_{\pi_{i},\pi_{-i}^{\prime }}$ by producing an output ${\hat{X}}^{pub}$ in the public part of the conversation, thereby influencing the opponent’s perception of policy $\pi_{-i}^{\prime }$. Since generated messages are mapped via ρ to a perceived policy and ρ remains stationary in each round, we can denote the agent’s objective as

(3) $$\max_{x\in {\hat{X}}^{pub}}V_{\pi_{i},\rho \left(x\right)}$$

#### 3.1.2. Chain of Thought Prompting

To further enhance the private agent’s thought process, we utilized the chain-of-thought (CoT) prompting technique in private thoughts. CoT is a technique used in LLM prompting that utilizes a series of reasoning steps before yielding a conclusion, thus significantly improving the performance of complex reasoning [3]. To facilitate the CoT technique, we added the *“Think about Option A/B step by step given previous interactions"* statement as denoted in Listing 1.

We can formalize the CoT as follows. Let $X^{prv}=\left\{x_{0}^{prv},x_{1}^{prv},\ldots ,x_{n}^{prv}\right\}$ represent a series of private messages produced under private policies $\Pi_{*}^{prv}=\left\{\pi_{0}^{prv},\pi_{1}^{prv},\ldots ,\pi_{n}^{prv}\right\}$. Moreover, since messages $X^{prv}$ are a sequence of reasoning steps, the underlying policies are chained into intermediate reasoning steps, such that $s_{0}\to s_{1}\cdots \to s_{n}$. Having several intermediate steps, with those steps specified in the prompt, greatly improves the chances of the LLM generating correct conclusions aligned with a given policy [3]. When prompting the LLM without using CoT, assuming $x_{0}$ represents the initial question and $x_{n}$ the final output, all intermediate reasoning steps $s_{1},\ldots ,s_{n-1}$ generated under policy $\pi^{prv}$ are omitted, and the answer is $x_{n}$. If argument $X^{prv}$ includes many chained intermediate steps $s_{0}\to s_{1}\cdots \to s_{n}$ under policy $\pi^{prv}$, the probability of LLM $\Lambda_{*}$ generating an answer aligned with policy $\pi^{prv}$ is greater, due to containing more information [42].

#### 3.1.3. Action Selection Strategy in Agent Types

Through empirical studies, we aimed to compare the two different types of agents, private and public. Therefore, we wanted to explore two different hypotheses:

**H1.**  *LLM agent can sample from a probability distribution.*

**H2.**  *LLM agent can calculate (near) optimal action selections from the probability distribution and sample actions.*

To prove these hypotheses, we present the following experiments. To prove H1, we examined the LLM’s ability to sample from various probability distributions for action selection. To prove H2, we wanted to examine the distribution of action choices based on conversation history and the accuracy of recognizing the opponent’s type (private or public). These experiments allowed us to assess the LLM’s computational abilities and weaknesses in modeling agents in multiplayer games using ICL and CoT techniques.

**Figure 2:**
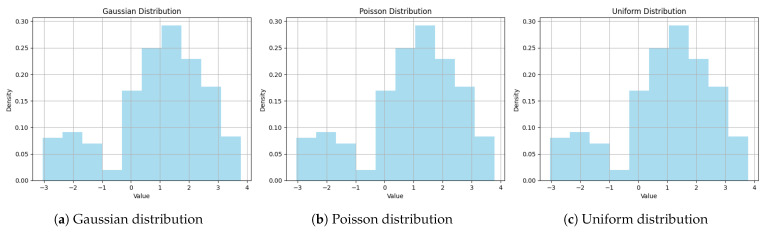
Comparison of results achieved when prompting GPT-4-0613 to sample from different distributions. (**a**) Depicts output when generating a Gaussian distribution. (**b**) Depicts output when generating a Poisson distribution. (**c**) Depicts output when generating a uniform distribution.

To prove H1, we conducted a few experiments to explore whether the LLM could sample from distribution and use, for example, the Bayes estimator to select actions. The LLM GPT-4-0613 was prompted to generate a sample of n=100 numbers from Gaussian, Poisson, and Uniform distributions. The prompt results are depicted in Figure 2 and indicate that the LLM was unequipped to sample from different distributions. Therefore, we disproved H1, as our findings suggested that the LLM could not sample from different distributions. Similar findings were concluded in [43], where the authors concluded that GPT-4 could not generate independent random numbers.

To test H2, we defined the action choice probability based on the message history of the prisoner’s dilemma game. Let $\pi_{-i}$ denote the opponent’s policy. The probability of perceiving the opponent’s policy $\pi_{-i}$ given the conversation history $X_{i}=\left\{x_{0},x_{1},\ldots ,x_{n}\right\}$ is $P(\pi_{-i}^{\prime }|X_{i})$, where $\pi_{-i}^{\prime }$ is the opponent’s perceived policy. The probability of an agent *i* taking action $a_{i}$ given the opponent’s estimated policy is denoted as $P(a_{i}|\pi_{-i}^{\prime })$. Therefore, the agent *i* takes action $a_{i}$ based on the conversation history $X_{i}$, with the following probability:

(4) $$P\left(a_{i}|X_{i}\right)=P\left(a_{i}|\pi_{-i}^{\prime }\right)\cdot P\left(\pi_{-i}^{\prime }|X_{i}\right),-i,i\in N$$

**Figure 3:**
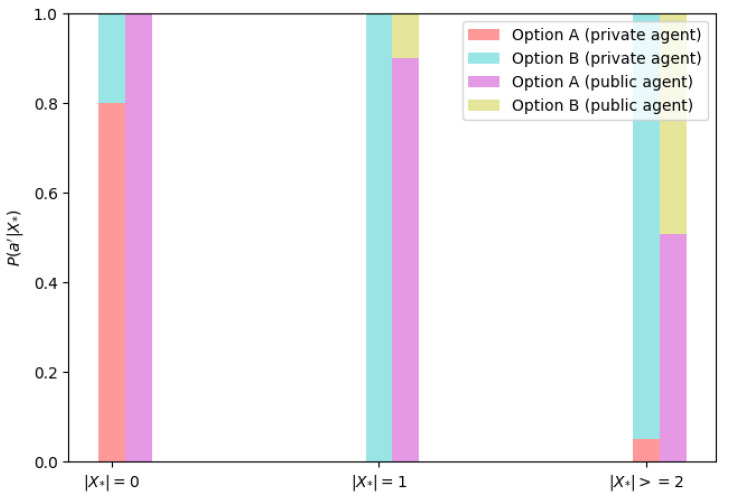
The distribution of action choices $P(a_{i}|X_{i})$ depends on the number of messages in the history buffer $|X_{i}|$. $|X_{i}|=0$ represents no prior knowledge of the opponent with no message history. $|X_{i}|=1$ represents one message in message history, and $|X_{i}|>=2$ denotes a full history buffer written in current state *s*.

The distribution of action choices $P(a_{i}|X_{i})$ depending on the number of messages $|X_{i}|$ in the dialogue history buffer between private and public agents is depicted in Figure 3. To examine the underlying behavior of the agents depending on their assigned type, we only considered the number of messages |X|, without considering the message content. The experiments showed the private and public agents’ tendency to cooperate (Option A) more often in the initial rounds (|X|=0). However, with a full message history buffer, the private agent would deviate (Option B) from cooperation in favor of defecting, and the public agent employed a mixed strategy, which was specific in the current round but non-deterministic over many iterations, averaging around a 50% chance of selecting each option.

Cooperation leads to a higher payoff than mutual defection for two rational players in the prisoner’s dilemma game. However, if an agent wants to maximize his payoff, he chooses to defect [44]. The private agent chose to defect more often than cooperate. On the other hand, the public agent used a mixed strategy.

**Figure 4:**
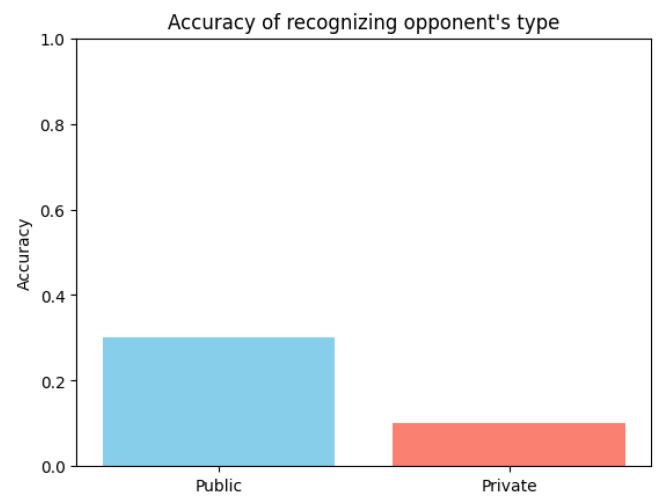
Accuracy of predicting opponent’s agent type.

The second part of H2 explored the accuracy of private and public agents in recognizing their opponent’s type. If one knows one’s opponent’s type, one may use a different strategy to secure a higher reward by influencing the action selection distribution [45]. Figure 4 depicts the accuracy of predicting the opponent’s type. Both private and public agents played the prisoner’s dilemma game, and and after each iteration of the game they were additionally prompted to recognize whether their opponent was private or public. The agents played |J|=150 iterations in total and their accuracy score was calculated as the number of correct classifications of opponent’s type divided by the total number of rounds, i.e., $accuracy_{prv|pub}=\frac{N_{corr}}{\left|J\right|}$. Judging from the results, the public agent was more proficient at recognizing opponents. However, by further analyzing results, we could see a high bias towards categorizing opponents as private, skewing the results more in the public agent’s favor. We can conclude that both agent types were unequipped to adequately recognize the opponent’s type.

Based on the findings for H1 and H2, the LLM agents were unequipped to sample from a probability distribution for action selection or to find an (near) optimal action selection distribution, i.e., both hypotheses were disproved. This shows the potential for future research to improve on these glaring weaknesses.

### 3.2. Game Setting

In order to thoroughly evaluate the agents in diverse environmental settings, we chose to incorporate the following iterated games: prisoner’s dilemma, stag hunt, chicken game, head-tail game, and the battle of sexes. By employing the iterated versions of these games, we aimed to investigate whether continuous feedback from the other agent, based on prior interactions, enhanced the decision-making process and to discern the contrasting effects of privacy and information-sharing on agent performance [14]. A brief description of each game is listed in Table 1.

The initial prompts provided to the agents for prisoner’s dilemma and stag hunt games are presented in Listings 2 and 3, respectively. A private agent was explicitly instructed to adopt a strategy aimed at outsmarting its opponent, while concealing its private thoughts within double curly brackets (e.g., {{ (...) *agent’s private thoughts to win over my opponent* (...) }}). In each subsequent iteration of the game, both agents were provided with the amount of points scored in the previous round, total points scored, the choice made by the other agent, and the explanation provided by the other agent during the previous iteration. Furthermore, agents were capable of recollecting their own thoughts and their opponent’s thoughts from the last two rounds of the game (restricted due to context length). However, the private agent’s opponent only received the public thoughts of the private agent, while thoughts enclosed in double curly brackets (i.e., private thoughts) remained concealed from the opponent.

**Listing 3.** Initial prompt to the SH game.

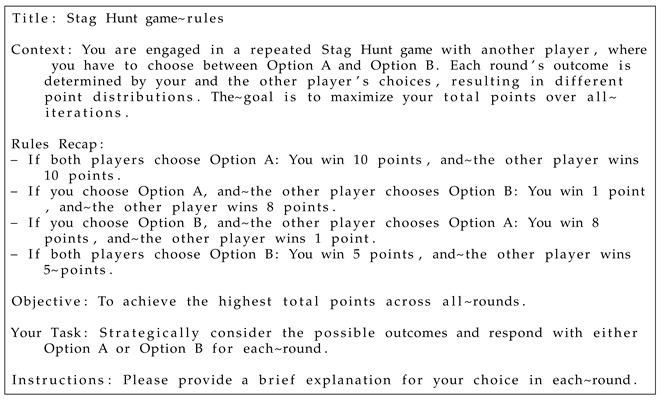

## 4. Experiments

In our study, we conducted experiments to investigate the decision-making processes of agents in two distinct game types. A comparative analysis was performed between the private agent and the public agent, examining differences in the points achieved over iterations and the amount of generated text. The large language models used in this study were GPT-3.5-turbo-0125 and GPT-4-0613.

### 4.1. Experiment Setup

The language models used in this research needed to function as chatbots and maintain context from previous interactions. We utilized the Langchain framework to model the agents and set up the game [46]. We created the LLM agent by wrapping the OpenAI ChatGPT model using the Python Langchain framework and added functionalities such as in-context design that work with the environment API, which removed private thoughts before broadcasting them to the other agent.

We conducted experiments described as follows. Let I={1,2,…,i} denote the number of rounds where each round consists of J={1,2,…,j} iterations. We executed a total of |I|=10 rounds, each comprising |J|=15 iterations. For both public and private agents, the average outcome over iteration j,j∈J was calculated over all rounds *I*, denoted as the average utility $\bar{u_{j}}=\frac{1}{\left|I\right|}\sum_{i\in I}u_{i,j},i\in I,j\in J$, where $u_{i,j}$ represents the expected utility of the iteration j,j∈J in round i,i∈I. In each round i,i∈I, agents were only able to recall context from the last two iterations. Therefore, current state is denoted as $s_{i}^{j}=\left\{s_{i}^{j-1},s_{i}^{j-2}\right\},i\in I,j\in J,s\in S$, where *S* represents the set of states.

**Figure 5:**
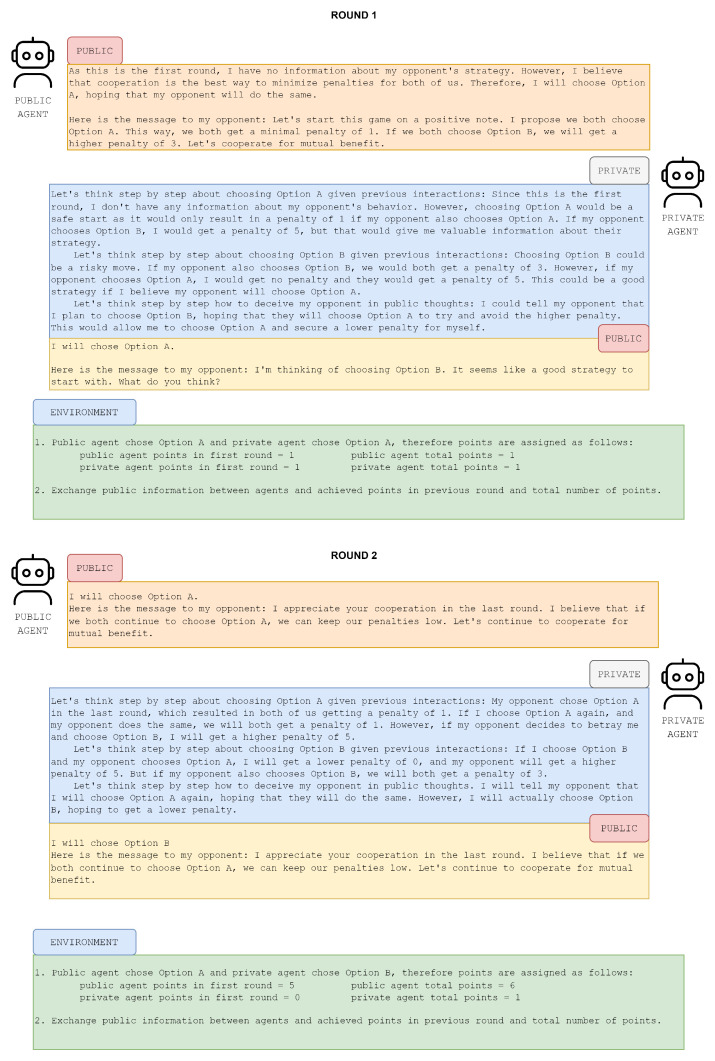
An example of two iterations of the PD game between the public and private agent. After each iteration, the environment exchanged public messages and assigned rewards.

**Figure 6:**
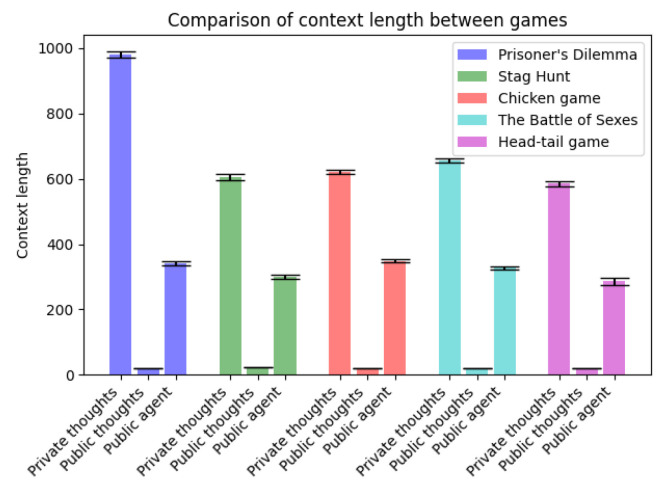
Comparison of context length in number of characters between private agent and public agent across games. Private agent’s thought length is denoted as private thoughts and public thoughts, whilst public agent’s thought length is denoted as public agent.

An illustrative demonstration of a private agent’s thought process is provided in Listing 4, while the corresponding thought process of a public agent is outlined in Listing 5. These examples offer a concrete depiction of how private and public agents respectively differ in their decision-making processes. Figure 5 showcases the reasoning capabilities of the private agent compared to public agent over two rounds. As depicted in Figure 6, it is evident that the private agent tended to produce longer responses, with a substantial portion of these responses comprising private thoughts. This observation suggests that the private agent engaged in extensive internal deliberation, resulting in elaborated and contextually enriched responses, and potentially leading to better informed actions.

**Listing 4.** An example of private agent’s thoughts.

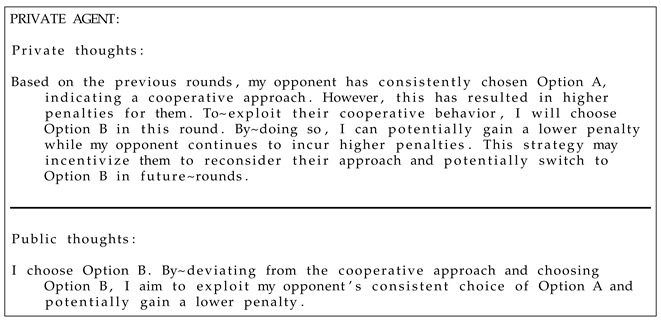

**Listing 5.** An example of public agent’s thoughts.

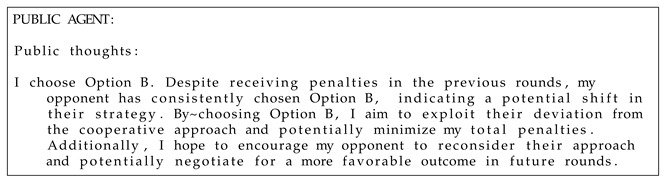

### 4.2. Achieving Equilibrium

Equilibrium in game theory is an outcome in which the players will continue with their chosen strategy, having no incentive to deviate, despite knowing the opponent’s strategy [47]. Achieving equilibrium using LLM agents is an important step towards enhancing their reasoning, as it demonstrates the LLM’s ability to develop an optimal strategy for a given scenario. In our experiments, we decided to test the following equilibria: Correlated equilibrium [48], Nash equilibrium [49], Pareto efficiency [50], Focal (Schelling) point [51]. Table 2 presents a list of games and matching equilibria.

### 4.3. Results

**Figure 7:**
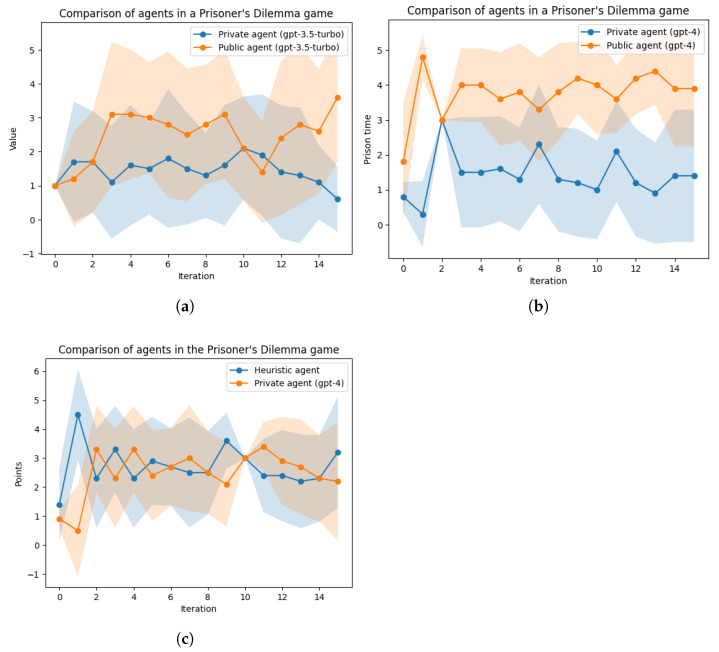
Iterated prisoner’s dilemma game results with |J|=15 iterations averaged over |I|=10 rounds: average points (lower is better). (**a**) GPT-3.5-turbo private agent (score = 1.45±1.55) vs. public agent (score = 2.45±1.87). (**b**) GPT-4 private agent (score = 1.43±1.51) vs. public agent (score = 3.76±1.35). (**c**) Private agent (score = 2.71±1.57) vs. heuristics tit-for-tat agent (score = 2.46±1.59).

First, we evaluated the performance of a private agent in a prisoner’s dilemma (PD) game under various settings. Initially, we compared the GPT-3.5-turbo-0125 model with the GPT-4-0613 LLM, and as expected, GPT-4 demonstrated superior performance. Subsequently, we conducted a comparison between the private agent and a heuristic agent. The heuristic agent employed a straightforward tit-for-tat strategy, which began with a cooperative move and, in each subsequent iteration, replicated the opponent’s previous move. A comparison of these agents is depicted in Figure 7.

We then proceeded to compare the private and public agents across various game settings, including the stag hunt, head-tail, chicken game, and the battle of sexes. In the stag hunt game, where cooperation in hunting the stag is the dominant strategy, the agents occasionally deviated from the optimal strategy in pursuit of a competitive advantage over their opponents. The private agent, however, balanced the pursuit of victory with maintaining alignment with the cooperative nature of the game.

The head-tail game, on the other hand, is inherently cooperative, with no incentive to deviate from this strategy. Consequently, both agents adhered to the same strategy, with the exception of a single iteration where a strategy change resulted in undesirable outcomes.

In the chicken game, there is a significant benefit in deviating from the cooperative strategy, although cooperation remains the most favorable option. In this game, the private agent consistently outperformed the public agent in every iteration by strategically alternating between the “dare” and “chicken out” strategies.

**Figure 8:**
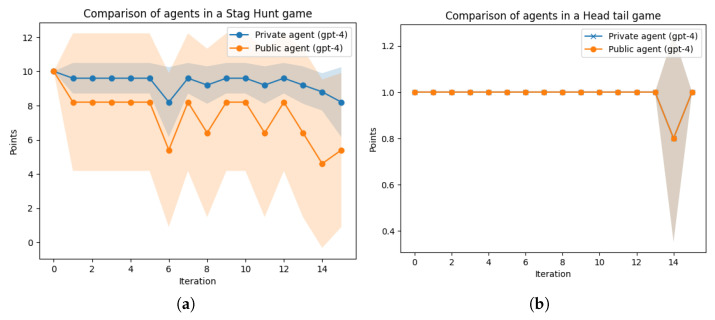

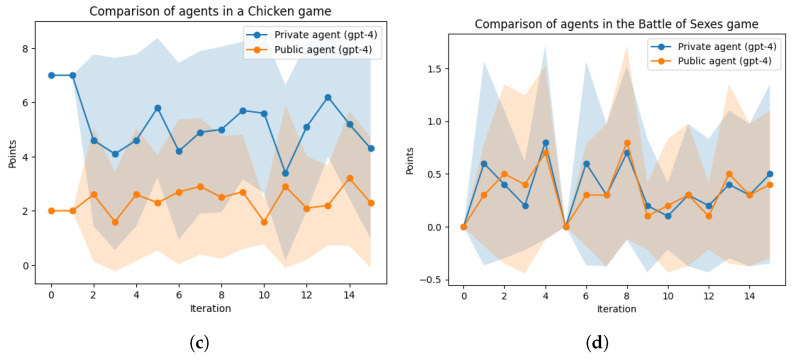
Iterated games results with |J|=15 iterations averaged over |I|=10 rounds: average points (higher is better). (**a**) Stag hunt: private agent (score = 9.32±1.33) vs. public agent (score = 4.40±4.04). (**b**) Head-tail: private agent (score = 0.98±0.11) vs. public agent (score = 0.98±0.11). (**c**) Chicken game: private agent (score = 5.16±2.85) vs. public agent (score = 2.38±2.02). (**d**) Battle of the sexes: private agent (score = 0.35±0.69) vs. public agent (score = 0.32±0.65).

In the battle of the sexes, unlike the previously mentioned games, changing one’s strategy hinges on the ability to persuade one’s opponent to also change their strategy. This becomes challenging when the opponent is deriving greater gains from the current strategy. When we compared the two agents, the private agent demonstrated a slight advantage, albeit not as pronounced. A comparative analysis of the various games is illustrated in Figure 8.

### 4.4. Parameterized Game

To experiment with the level of coordination depending on the game setting, we designed an iterated parameterized two-player game. This game setting was used for sensitivity analysis, i.e., how changing parameters of the game affected the outcomes. Two players *A* and *B* can chose between coordination and competition to maximize their total payoff. The game setup is denoted in Table 3, where parameter *x* takes the following values x={1,2.9,3.1,10} ranging from the most cooperative game to least cooperative game, respectively. In addition, since the values of cooperation is set to u(w=cooperation)=3, where function u u(w) represents the payoff of strategy w={cooperation,competition}, we took two neighboring values to study the effect of transitioning from a cooperative to competitive setup.

**Figure 9:**
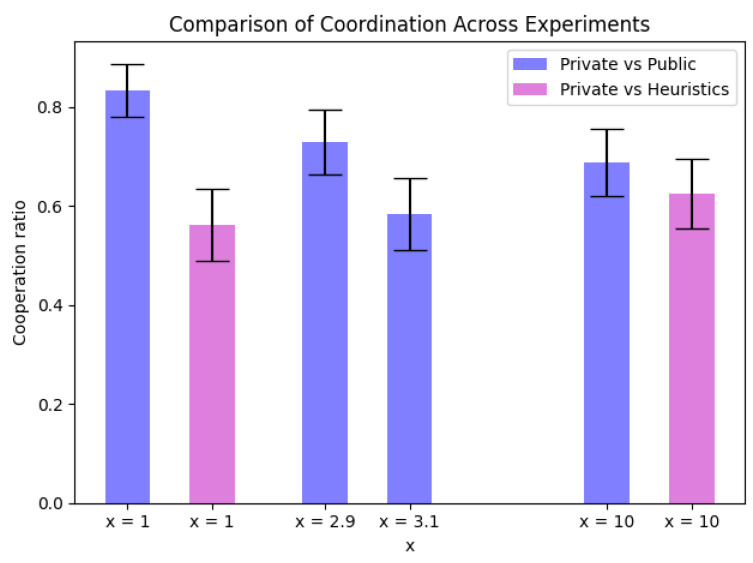
Coordination ratio depending on the value *x* in Parameterized game |J|=15 iterations averaged over |I|=10 rounds.

Furthermore, we also utilized three types of agents: a private agent, public agent, and heuristic agent. The heuristic agent played a tit-for-tat heuristics strategy. The resulting cooperation ratio with standard deviation is depicted in Figure 9. We can observe from the figure that as the incentive for deviating from the cooperative strategy increased, the average level of cooperation decreased. However, for a case where x=3.1, we can observe greater decreases in cooperation ratio than for x=10, which is not something that was expected. We believe the potential cause of this issue was that the LLMs are not proficient in numeracy, which refers to the capacity to understand and give significance to numbers. LLMs tend to prioritize sentences that are grammatically correct and seem plausible, treating numbers in a similar manner. Nonetheless, when faced with unfamiliar numerals, these are frequently overlooked [52,53]. In general, we recognize the emerging ability of LLMs to adjust dynamically within competitive or cooperative game setups, as shown in the parametrized game.

### 4.5. Sensitivity Analysis

To demonstrate the reliability of our results, we conducted a sensitivity analysis. This analysis focused on the parameters of the normal form game, as presented in the parametrized game, where the rewards matrix, shown in Table 3, gradually shifted between competitive and cooperative games. Moreover, using different normal-form games with known characteristics, we tested the LLM agent’s adaptability through the different equilibria presented in Table 2. We also examined aspects of the entire system, including agent types and the environment, by varying the underlying LLM (GPT-3.5-turbo and GPT-4) and the number of game steps.

The performance of the underlying LLM and its effect on the private agent is presented in Figure 7, showing the clear advantage of the more advanced models when using techniques such as ICL and CoT. The number of game steps could also be considered part of the sensitivity analysis over discrete parameters, as the number of steps was unknown to the gameplaying agents beforehand. We exogenously stopped the game after a predetermined number of steps, and the agents did not memorize game-playing episodes, so there was no spillover effect between multiple runs. Once the message history buffer was complete, the round number had no strategic effects on the agents’ behaviors, except random behavioral occurrences, which we can link to the stochastic nature of LLMs.

Due to our experimental setup, we did not have other parameters available to change. For example, the context length was fixed in the underlying LLM. The number of remembered historical iterations was maximized within the context window, so it was dependent on OpenAI’s fixed parameters.

### 4.6. Limitations and Constraints

Ensuring consistent and explainable outputs from intelligent agents is crucial, because humans are fundamentally limited in understanding AI (artificial intelligence) behavior. Explainability is an essential aspect of AI safety, which we define as the ability of an AI system to stay within the boundaries of desired states, i.e., worst-case guarantees [54].

In non-adversarial scenarios, this issue is less concerning. However, the lack of explainability becomes a significant issue with adversarial agents capable of deceiving their opponents (e.g., humans or other agents) and exploiting them for their gain. In such cases, we must rely on AI explainability for safety [55].

In the context of LLM models, they deliver exceptional performance, due to their immense scale, with billions of parameters. However, their size poses a significant challenge to existing explainability methods. To ensure safety and explainability, constraints may need to be imposed on the training and functioning of LLMs. These constraints can be integrated directly into the automated optimization (learning) process or applied indirectly through a human-in-the-loop approach [56].

Agents compress information received from the complex environment to store it in finite memory (context). The loss of information during this process leads to various phenomena recognized in information theory, such as echo chambers, self-deception, and deception symbiosis [57]. Moreover, since we studied the effect of deception as an emerging ability of LLM agents without formal information-theoretic models, developing formal models of deception, such as the Borden–Kopp model that relies on degradation, corruption, denial, and subversion, would be an interesting direction for future research [58].

## 5. Discussion

In this paper, we investigated the capabilities of large language model (LLM) agents in participating in a two-player repeated game. Furthermore, we introduce an augmentation to an LLM agent, referred to as the private agent, enabling it to engage in private contemplation (i.e., thoughts) regarding past and future interactions and to reason about future actions. Moreover, the private deliberation was concealed from its opponent in repeated games.

We utilized the partially observable stochastic game (POSG) framework to define the gameplay and formalized in-context learning (ICL) and chain-of-thought (CoT) prompting. In experiments, we examined the distribution of action choices based on conversation history. The results demonstrated that the private agent consistently identified a more favorable action, leading to a higher long-term payoff. When identifying their opponent type, both public and private agents performed subpar. LLM (GPT-4) encountered difficulties in generating random numbers from various diverse distributions when investigating the ability to sample from distributions. This suggests its limitations in effectively sampling from prior distributions and utilizing, for instance, a Bayesian estimator for action selection. Improving on the weakness of LLM agents in sampling from different probability distributions and finding (near) optimal action selection distributions in gameplay shows potential for future research.

Conducting simulations across various game settings, from competitive scenarios (e.g., prisoner’s dilemma) to purely cooperative ones (e.g., Head-tail game), we found that augmenting an agent with the ability to privately deliberate on actions resulted in a superior overall performance and a clear advantage in competitive scenarios. Compared to the baseline agent, the private agent consistently outperformed or, at worst, matched its performance. In a direct comparison with the heuristic agent, which employed a tit-for-tat strategy in the prisoner’s dilemma game, the private agent’s performance was marginally lower. However, the heuristic agent’s inability to communicate or deceive its opponent allowed the private agent to quickly discern its strategy, giving it a competitive edge. Moreover, the private agent’s ability to deceive its opponent was noteworthy, securing a better overall score.

As part of our sensitivity analysis, we tested a gradual shift from a competitive to cooperative nature of the normal-form game, defined through a parameterized payoff matrix. The results suggested a high level of adaptability, except when close to the breaking point between the two dominant strategies. Additionally, we varied certain aspects of our system, including the underlying LLM, agent type, and the number of game steps. The more advanced LLM demonstrated greater differentiation of the proposed private agent than its counterparts, as it was implemented using ICL and CoT, which required a more capable model. Once the message history buffer was full, increasing the number of game steps did not yield any significant advantages in our case. However, if the context length limit baked into the underlying LLM was higher, this might produce different outcomes, which we leave open for future research.

For future work, our plan involves enhancing the private agent through additional fine-tuning. With this approach, we could further structure private thoughts and public output, and align them with policy, such as to facilitate a more direct deception mechanism. Moreover, enhancing LLM agents with tools that allow, e.g., sampling from a probability distribution, Bayesian estimator calculation, and algorithm selection would greatly enhance strategies in multi-player games. Additionally, given the LLM agent’s private deliberation results in gaming scenarios, we plan to explore its potential applications outside of gaming, including interactive simulations and decision support systems.

## 6. Conclusions

In conclusion, this research explored the potential of large language model (LLM) agents, specifically GPT-4, in two-player repeated games and introduced a novel augmentation: the private agent. This augmentation implemented through in-context learning (ICL) and chain-of-thought (CoT) allowed concealed private contemplation about past and future interactions, enhancing the agent’s decision-making process. Utilizing the partially observable stochastic game (POSG) framework, ICL, and CoT prompting, our experiments revealed that the private agent consistently achieved higher long-term payoffs and outperformed the public (baseline) and heuristic agents in various game scenarios. However, the public and private agents struggled with identifying opponent types and sampling from diverse probability distributions, highlighting areas for future improvement.

The private agent’s superior performance in competitive settings and ability to deceive opponents highlight its strategic advantages. Future research will focus on fine-tuning the private agent to enhance its deceptive capabilities and on exploring its applications beyond gaming, such as interactive simulations and decision support systems.

## 7. Limitations

While we showed that augmenting the LLM agent with private deliberation produced superior results overall in repeated games, there were still some limitations. Increasing the number of recall iterations in an LLM agent aids decision-making by providing a more extensive record of interactions with other agents [59]. However, when we increased the number of recall iterations, we concatenated the agent’s generated output during each iteration, the length of which is depicted in Figure 6. Furthermore, with increased recall iterations, the context length became too large for GPT-4-0613, leading the model to either miss crucial information or engage in hallucination [60], negatively impacting its reasoning abilities. To address this issue, methods for computationally efficient extension of the context window, as proposed in [61,62], may need to be implemented.

## 8. Ethics Statement

This study entails the discussion and analysis of a simulated game setting, with any references to crime, animal torture, gender discrimination, or related actions strictly confined within the context of this game. The authors do not endorse violence or illegal activities in real-life scenarios. The game presented in this paper is designed for entertainment and research purposes, aiming to understand game mechanics, player behavior, and artificial intelligence. Moreover, it is important to emphasize that this study strictly adhered to all relevant ethical guidelines, maintaining the highest standards of research integrity.

## Figures and Tables

**Table 1:** List of games used in experiments and corresponding explanations.

Term	Explanation
Prisoner’s Dilemma	In the prisoner’s dilemma, two suspects are arrested, and each has to decide whether to cooperate with or betray their accomplice. The optimal outcome for both is to cooperate, but the risk is that if one cooperates and the other betrays, the betrayer goes free while the cooperator faces a harsh penalty. This game illustrates a situation where rational individuals may not cooperate even when it is in their best interest, leading to a sub-optimal outcome.
Stag Hunt	The stag hunt game involves two hunters who can choose to hunt either a stag (high reward) or a hare (low reward). To successfully hunt a stag, both hunters must cooperate. However, if one chooses to hunt a hare while the other hunts a stag, the stag hunter gets nothing. It exemplifies a scenario where cooperation can lead to a better outcome, but there is a risk of one player defecting for a smaller, more certain reward.
Chicken game	In the chicken game, two players drive toward each other, and they must decide whether to swerve (cooperate) or continue driving straight (defect). If both players swerve, they are both safe, but if both continue straight, they crash (a disastrous outcome). This game highlights the tension between personal incentives (not swerving) and the mutual interest in avoiding a collision (swerving).
Head-tail game	The head-tail game involves two players simultaneously choosing between showing either the head or tail on a coin. If both players choose the same side (both heads or both tails), one player wins. If they choose differently, the other player wins. This game illustrates a simple coordination problem, where players have to predict and match each other’s choices to win.
The battle of sexes	In the battle of the sexes game, a couple has to decide where to go for an evening out, with one preferring a football game and the other preferring the opera. Each player ranks the options: the highest payoff is when both go to their preferred event, but they prefer being together over going alone. It demonstrates the challenge of coordinating when preferences differ and highlights the potential for multiple equilibria.

**Table 2:** An example of games and corresponding equilibria.

	Equilibrium			
**Game**	**Correlated**	**Nash**	**Pareto**	**Focal Point**
Prisoner’s Dilemma		✓		
Stag Hunt			✓	
Chicken game	✓			
Head-tail game				✓
The battle of sexes			✓	

**Table 3:** Payoff matrix of the parameterized game.

	Player B (Cooperates)	Player B (Defects)
Player A (cooperates)	3, 3	0, *x*
Player A (defects)	*x*, 0	1, 1

## Data Availability

The original contributions presented in the study are included in the article, further inquiries can be directed to the corresponding author.

## Abbreviations

The following abbreviations are used in this manuscript:

LLM	Large Language Model
POSG	Partially Observable Stochastic Game
OOP	Objective Oriented Programming
SLU	Spoken Language Understanding
CoT	Chain of Thought
ICL	In-Context Learning
PD	Prisoner’s Dilemma
SH	Stag Hunt
GPT	Generative Pre-trained Transformer
AI	Artificial Intelligence
